# Transforming growth factor beta-1 (TGF-β1) expression in patients with adenomyosis

**DOI:** 10.61622/rbgo/2024rbgo31

**Published:** 2024-04-09

**Authors:** Andreia Jacobo, Renata Fogaça Borges, Carlos Augusto Bastos de Souza, Vanessa Krebs Genro, João Sabino Cunha-Filho

**Affiliations:** 1 Universidade Federal do Rio Grande do Sul Porto Alegre RS Brazil Universidade Federal do Rio Grande do Sul, Porto Alegre, RS, Brazil.

**Keywords:** Adenomyosis, Etiopathogenesis, Immunohistochemistry, Transforming growth factor β1

## Abstract

**Objective:**

To compare Transforming growth factor beta-1 (TGF-β1) expression in patients with and without adenomyosis.

**Methods:**

A prospective design was performed including 49 patients submitted to hysterectomy. Immunohistochemistry was performed on anatomopathological samples staged in paraffin blocks from patients with and without adenomyosis. The sample contained 28 adenomyosis cases and 21 controls. Student's *t*-test and multivariate logistic regression tests were used for statistical analysis. Associations were considered significant at *p* < 0.05.

**Results:**

We found no significant association between adenomyosis and: smoking (*p* = 0.75), miscarriage (*p* = 0.29), number of previous pregnancies (*p* = 0.85), curettage (*p* = 0.81), pelvic pain (*p* = 0.72) and myoma (*p* = 0.15). However, we did find a relationship between adenomyosis and abnormal uterine bleeding (AUB) (*p* = 0.02) and previous cesarean section (*p* = 0.02). The mean TGF-β1 intensity (mean ± SD) in the ectopic endometrium of women with adenomyosis showed no significant association (184.17 ± 9.4 vs.184.66 ± 16.08, *p* = 0.86) from the topic endometrium of women without adenomyosis.

**Conclusion:**

TGF-β1 expression was not increased in the ectopic endometrium of women with adenomyosis.

## Introduction

Adenomyosis is a common gynecological disease; its prevalence varies from 5 to 70% in symptomatic women, with a mean frequency of 20 to 30% in hysterectomy specimens.^(1)^ The symptoms associated with the disease are menorrhagia, dysmenorrhea, and metrorrhagia, and one-third of patients are asymptomatic.^(1,2)^ Hysterectomy is the treatment of choice for the disease because clinical treatment yields poor results and high recidivism rates after discontinuation.^(3,4)^

The topic and ectopic endometria of women with adenomyosis have shown a number of metabolic and molecular changes: increased angiogenesis and proliferation; decreased apoptosis, enhanced local estrogen production, progesterone resistance, and undermine cytokine expression. These changes increase the endometrium's ability to infiltrate the myometrial junctional zone and facilitate the growth of ectopic tissue. Furthermore, several immunological abnormalities have been observed in these individuals, together with an increase in "free radical" production, which leads to the overgrowth of endometrial stromal cells. This process may facilitate the establishment of adenomyosis.^(5)^ Metabolic and molecular abnormalities have also been observed that are often similar to those observed in endometriosis, where there is abnormal gene expression, local production of estrogen with an altered endometrial response to progesterone and an increase in nerve density and oxidative stress.^(6)^

Recently, we demonstrated^(7)^ that P-element-induced wimpy testis-2 (PIWI2) protein was over-expressed in patients with diffuse adenomyosis, this protein is involved in cellular survival and growth. However, PIWI1 protein is down-regulated which could be linked to the loss of cellular control or a hostile (ectopic tissue) environment.^(7)^

Transforming growth factor β (TGF-β) is abundantly expressed in the endometrium and is secreted by the endometrial cells and macrophages into the uterine fluid, where its interactions with pre-embryonic implantation are suspected.^(8)^ TGF-β secretion in the peritoneal fluid of women suffering from endometriosis suggests that it may be critical in the establishment and/or maintenance of endometriosis.^(9)^

TGF superfamily members promote their biological responses by binding to transmembrane serine/threonine kinases, known as TGF superfamily receptors.^(10-12)^ In a study aimed to determine whether leiomyoma, adenomyosis, and endometrial polyps are associated with changes in matrix metalloproteinases (MMP-2 and MMP-9) and cytokines in the endometrial cavity, it was noted that the TGF-β1 levels were significantly higher than those of the control group.^(13)^

Another study found that adenomyotic lesions had significantly higher platelet aggregation and increased expression of TGF-β1 and phosphorylated Smad3 than the control group.^(3)^ Understanding the role of TGF-β1 in adenomyosis could be important for a future medical treatment of this disease.

Because adenomyosis is very prevalent and has rather an obscure pathophysiology with some evidence pointing to cell invasion abnormality, we propose a study to evaluate TGF-β1 expression in the ectopic endometrium of women with adenomyosis and to compare it with that in the endometrium of patients without this condition, considering the role played by TGF-β1 in cell proliferation and tissue remodeling.

## Methods

This case-control study used tissue samples embedded in paraffin and fixed in 10% buffered formalin, which were taken from women who underwent a hysterectomy between the years 2013 and 2020.

This was a retrospective case-control database study. We included all hysterectomy specimens (n=145). Therefore, we divided cases: patients with diffuse adenomyosis who were not in menopause at the time of surgery, underwent surgery for benign disease and had no history of endometriosis (n=28), and hysterectomy specimens from patients undergoing surgery for a benign disease who had no diagnosis of adenomyosis or endometriosis were used as controls (n=21). Fifty patients were excluded because they presented one or more combined diagnosis: endometriosis (n=43), endometrial neoplasia (n=21), endometrial hyperplasia (n=13), postmenopausal status (n=9) and focal adenomyosis (n=9). Moreover, one patient was excluded from the analysis for insufficient anatomopathological material.

The study cases included the hysterectomy specimens of patients with diffuse adenomyosis who were not in menopause at the time of surgery, underwent surgery for benign disease and had no history of endometriosis. Hysterectomy specimens from patients undergoing surgery for a benign disease who had no diagnosis of adenomyosis or endometriosis were used as controls. Clinical data (age, weight, skin colour, number of pregnancies, cesareans, curettage, smoking, menopause, abortion, surgical indication, abnormal uterine bleeding (AUB), fibroids, operative route, pelvic pain, uterine volume by transvaginal ultrasound (TVUS), and specimen weight) were collected from medical records. Missing data were not replaced. The original hematoxylin and eosin slides were reviewed, and a paraffin block was selected for each case and for each control (areas with adenomyosis for the cases and topic endometrium for patients without adenomyosis). Samples with insufficient tissue for immunohistochemical staining were excluded. The final sample contained 28 adenomyosis cases and 21 controls for other diseases (fibroids, prolapse, or endometrial atrophy). The sample size was calculated using the article from Bergholt et al.^(14)^ We used the 18% prevalence of adenomyosis with a 95% confidence level and a 5% confidence interval in Creative Research Systems Survey Software (Sebastopol, CA, USA). The calculated sample size was 17.

The specimens (fixed in 10% buffered formalin, processed, and embedded in paraffin) were subjected to histological sectioning using a microtome set to a thickness of 4 µm. The sections were placed on previously slides. To perform the immunohistochemistry technique, the slides were heated in an oven at 80 °C for 30 minutes, deparaffinized in xylene, and rehydrated in ethanol and then in distilled water. Antigen retrieval was performed in a water bath for 20 minutes at 95 °C in citrate buffer pH 6.0. Endogenous peroxidase activity was blocked with 5% hydrogen peroxide solution in methanol for 20 minutes. The sections were then incubated overnight in a refrigerator at 2 to 8°C with the primary antibody (anti-TGF-β, Novusbio, clone: 7F6) at a 1:200 dilution. After incubation, the Santa Cruz Goat anti-mouse IgG-HRP, code SC2005 detection system was added at a 1:200 dilution and incubated for 1 hour and 30 minutes. Reaction visualization was obtained with a Liquid Diaminobenzidine (DAB) Kit (Dako, K3468, Agilent Technologies, Santa Clara, CA, USA), according to the manufacturer's recommendations. After visualization, the slides were counterstained in Harris hematoxylin for 10 seconds and differentiated in 2% ammonia water for 30 seconds. The sections were then dehydrated in absolute alcohol and placed in xylene to mount the slides in Entellan-type resin.

The stained sections were examined under an Olympus BX 51 microscope (Olympus Optical Co., Tokyo, Japan) connected to a QColor 5 colour digital camera (Olympus). The images were obtained with a 200-fold magnification under standardized lighting conditions. Each slide was coded and analyzed using ImageJ Software v.4.0.1 (available at http://rsbweb.nih.gov/ij/). The region of interest consisted of an area containing ectopic endometrium in the cases and topic endometrium in the controls, which was subjected to the analysis procedure known as "colour deconvolution", written as a "plugin" for the ImageJ software, using the panel with the DAB image. The final DAB intensity was calculated according to the following formula: ƒ = 255-i, where ƒ = final DAB intensity and i = mean DAB intensity obtained from the software. The mean DAB intensity varied from zero (white, expressionless) to 255 (dark brown, the highest expression).

IBM SPSS Statistics software version 20.0 (IBM, Armonk, NY, USA) was used for all statistical calculations. Student's *t-test* was used to compare the TGF-β1 expression (mean DAB value after application of the correction formula) and the variables age, smoking, menopause, gestation, cesarean section, uterine curettage, abortion, pelvic pain, abnormal uterine bleeding and fibroids in the endometrium of the two groups. We performed a series of multivariate analyses, such as logistic regression controlling adenomyosis with the expression of TGF-β1 through immunohistochemistry technique. In addition, we controlled the variables gestation, abortion, curettage, cesarean section and smoking with the presence or absence of adenomyosis. Associations were considered significant at *p* < 0.05.

This research protocol was approved by the institutional ethical committee 993076 (IRB, #40213114.5.0000.5327), as a retrospective data-base analysis, the informed consent form was not necessary.

## Results

**Figure 1:**
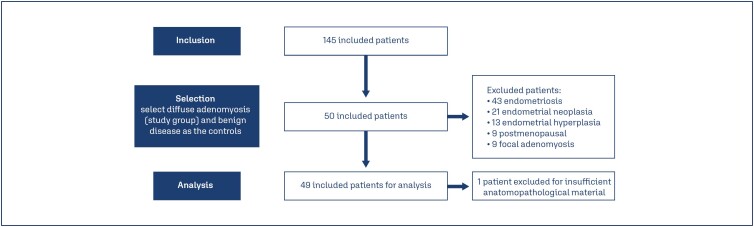
Analysis of criteria

One hundred forty-five patients were evaluated in this study but, we included 49 uterine specimens of patients who underwent hysterectomy between 2013 and 2020 and met the inclusions criteria, as demonstrated in the flowchart, for analysis (Figure 1).

The mean age was 46.64 ± 4.1 years (mean ± SD) for the adenomyosis group and 49.5 ± 7.03 years for the without adenomyosis group. The results of the study showed no significant associations between adenomyosis and the demographic and clinical variables presented in table 1.

**Table 1:** Characterization of patients from both groups that participated in the study

Variables		Without adenomyosis (n = 21) n(%)	With adenomyosis (n = 28) n(%)	p value*
Age (years)		49.5 ± 7.03	46.6 ± 4.1	0.79
Smoker		3(14.2)	4(14.2)	0.75
Menopause		0(0.0)	14(51.9)	0.001
Pregnancies		21(100)	28(100)	0.85
Cesarean section		4(19.0)	16(57.1)	0.02
Curretages		15(71.4)	22(78.5)	0.81
Abortions		10(47.6)	11(39.2)	0.29
Pelvic pain		7(33.3)	8(28.5)	0.72
Abnormal uterine bleeding		13(61.9)	27(96.4)	0.02
Uterine myomatosis		10(47.6)	19(67.8)	0.15
Operative route				
	Abdominal	3(14.2)	5(17.8)	0.03
	Vaginal	9(42.8)	3(10.7)	
	Videolaparoscopic	9(42.8)	20(71.4)	

Variables’ analysis between both groups taking into account its distribution and frequencies. Continuous variables are described as mean ± standard deviation (SD) and categorical variables are described as absolute and percentile frequencies, respectively, n(n%)

**Figure 2:**
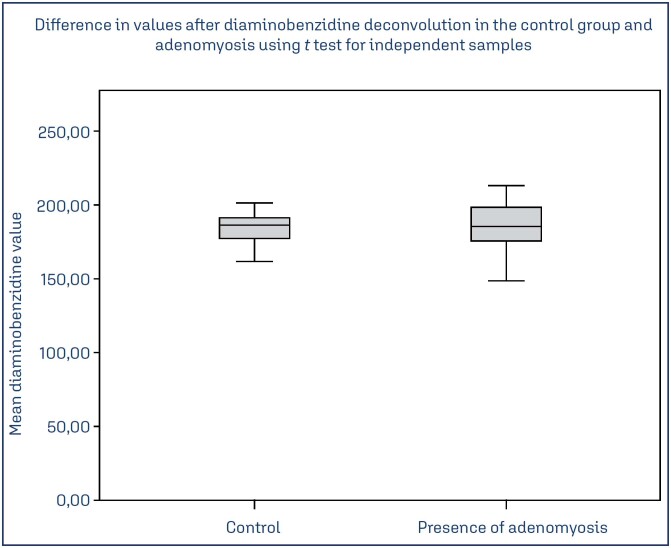
Difference in values after deconvolution between the groups with and without adenomyosis

**Figure 3:**
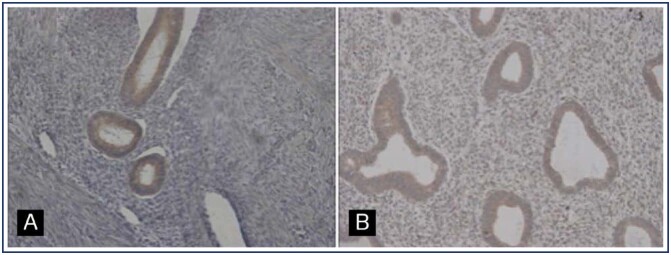
Immunostaining of TGF-B1 in adenomyosis (A) and the control endometrium (B)

Therefore, adenomyosis was related to abnormal uterine bleeding (AUB) (*p* = 0.02) and previous cesarean section (*p* = 0.02). Regarding the transforming growth factor (TGF-β1) expression, we found no significant association between the ectopic endometrium of women with adenomyosis and the topic endometrium of patients without this condition (*p* = 0.86) (Figures 2 and 3).

Furthermore, the linear logistic regression was applied between the groups controlling adenomyosis with the results of immunohistochemistry and we did not find an association between protein expression of TGF-β1 with the presence or absence of adenomyosis (p = 0.83), which confirms our previous findings. We did the same multivariable analysis including age, tobacco use, c-section, abnormal uterine bleeding, myomatosis, menopause status and previous obstetric history. All included variables were not related to the protein expression of TGF-β1.

## Discussion

We presented that TGF-β1 expression between the ectopic endometrium from adenomyosis patients and topic endometrium from the control group was not different.

Adenomyosis is a complex disease with several phenotypes, consequently, the extrapolation of all results is difficult and we should include as homogeneous as possible our group of patients. For this reason, we included only diffuse adenomyosis without endometriosis. Moreover, this disorder could be divided (physiopathology) into TGF-dependent and TGF-independent.^(15)^ This difference could be associated with a dysfunctional myofibroblasts population, some authors described also microtrauma in the endometrial/myometrial junctional zone as an important event in patients with adenomyosis.^(16)^

However, another author studying 18 patients showed that TGF was present and with an altered expression in the smooth muscle in adenomyosis species.^(17)^ This result was reinforced by an inhibition of TGF using a mouse model of adenomyosis and an anti-TGF treatment, the anti-inflammatory effect of TGF was reversed and this opened a new perspective in terms of future treatment of this disease.^(18)^ Moreover, in the future if we differentiate diffuse adenomyosis TGF positive x negative, the treatment could be different, using for example an anti-TGF target drug.

Inagaki et al. evaluated the cytokine expression in uterine flushings from patients with adenomyosis, leiomyoma, and uterine polyps and found significantly higher TGF-β1 levels than in the control group.^(13)^ In another study, using the same methodology, Liu et al.^(3)^ observed a significant increase in platelet aggregation, TGF-β1 expression, and phosphorylated Smad3 in patients with endometriosis and adenomyosis compared with the control group.^(3)^

Our findings are in agreement with others^(16)^ that did not demonstrate al altered TGF expression in the 32 analyzed patients. Furthermore, although TGF-β1 did not correlate with adenomyosis, our result showed no indication that TGF-β1 is or is not associated with the genesis of the disease. Early in its development, TGF-β1 may be associated with adenomyosis in facilitating cell growth and invasion. However, our study shows that it has no function in the late maintenance of the disease; we cannot refute that TGF-β1 could be important for cell proliferation and tissue remodeling in the genesis of adenomyosis. In addition, we cannot exclude the junctional zone micro-trauma and even the different physiopathology of this disease involving TGF-independent/TGF-dependent.

he adenomyosis cases. This result is explained by the fact that we performed the analysis on hysterectomy specimens, and as mentioned earlier, data in the literature suggest that the presence of sex steroids, both estrogen and progesterone, positively affect TGF-β1 expression. Another limitation is the characterization of adenomyosis, which may present in focal (adenomyoma) or diffuse form. In our study, we did not use hysterectomy sections with diagnosed adenomyoma or focal adenomyosis, only diffuse form was included.

We could include also the endometrial tissue from adenomyotic patients; however, the main objective and most relevant information for determining the effect of TGF-β1 in adenomyosis was to compare the topic endometrium (control group) versus the adenomyosis tissue (ectopic tissue).

In conclusion, diffuse adenomyosis in older patients was not associated with TGF-β1, but we should stress that this disease is not homogeneous and is composed of several phenotypes having a different fibrogenic mechanisms.

## Conclusion

We concluded that TGF-β1 expression was not enhanced in the ectopic endometrium of women with adenomyosis.
